# Notes and Comments

**Published:** 1893-12

**Authors:** 


### Notes and Comments.[1]

"THE atmosphere we create has so keen an effect on those about us that our personal comments are, after all, but a reflection of ourselves. It is the jealous temperament that discovers defects; the critical temperament that sees all the faults; the provincial mind that carries the non-elastic six-inch rule to measure giants and pigmies; the generous mind that sees all perfection; the discriminating mind that sees the whole man."

THE TEST OF STRENGTH.—The following quotation, taken from a recent editorial in *The Outlook*, suggests some thoughts for the dentist. The writer, in speaking of women (though they be college graduates), often feeling as if their lives were limited by the petty details to which the mind of the housekeeper must give attention,

[1] The assistant editor solicits contributions for this department,—new methods, new remedies and formulas, or any short practical note which may prove of value to the practitioner or student. Address 1506 Arch Street, Philadelphia.

says, "The every-day detail inseparable from the administration of a household, large or small, makes the sum total of its mistress's happiness. To neglect or overlook the smallest detail for one day means double care, or increase of friction, for the days that follow. It is the omission of the pinch of salt that spoils the dinner. It is the care of the trifling things, the small essentials, that marks the difference between a well-organized and a disorganized home."

Now, to apply this to ourselves. Every one must learn that it is the care and nicety of attention with which details are met that makes the difference between well-applied intelligence and ignorance, or its equivalent, indifference. It is our ability to attend to details perfectly, that is, in order and without producing friction, or to train others to attend to them in the same spirit, which marks the difference between the successful and the unsuccessful man.

In our colleges too, this matter of details is of greater importance than is usually accorded it. Teachers sometimes fail to make their work a success in that they slight the little things. By giving more attention to the details, students are better able to grasp the subject, comprehend the teaching, and they make more practical men, thus doing greater credit to the school.

Not infrequently are colleges and universities compared according to the appearance of their buildings, and the amount of noise they make in the community. It is too often forgotten that schools of any kind are only valuable to the world in what they produce.

---

Drilling Cavities in Artificial Teeth.—Dr. E. T. Davis, of Bridgeton, N. J., writes us upon this subject as follows: "Many of us, not being convenient to the dental depots, should be able to prepare the cavities ourselves. I find this can be done very nicely by using an inverted cone or wheel drill (on the engine), which must be kept wet with spirits of turpentine. In this way the cavity, under-cuts, and retaining pits can be made without any trouble."

While this, in the hands of some, may give good results, the ordinary diamond stone and drill will give more universal satisfaction.

---

Pyorrhœa Alveolaris—An Important Announcement.—At the regular meeting of the New York Odontological Society, held November 21, 1893, Professor C. N. Peirce, of Philadelphia, read a paper on the "Etiology of Pyorrhœa Alveolaris," in which he stated that, in the effort to reduce this disease to its simplest factors and determine the primary origin of each, he should coin two terms, which he thought would be more expressive as to the true nature of the disease. He believed that in one form of calcic pericementitis the origin of the salt was the saliva, and in the other form, the blood; the former he therefore designated as ptyalo-genic calcic pericementitis, expressing the idea that in its origin it is local, peripheral, and salivary; the latter he designated as hæmato-genic calcic pericementitis, expressive of the idea that in its origin it is constitutional, central, and associated with some modification of the normal composite of the blood-plasma. This latter he believed was the condition in true pyorrhœa. In this he suggested that possibly some chemical agent derived from the blood, the product of some morbid constitutional state, might be the exciting cause. With the view of testing the plausibility of this assumption, he had the deposit removed from the apical extremities of teeth which had been sacrificed by this so-called pyorrhœa, and subjected to chemical analysis. In every instance the chemical methods employed revealed the fact that the deposit was a combination of calcic urate, sodic urate, with some calcic phosphate and carbonate. *The existence of the urates, in which the uric acid is the predominating element, shows that this deposit is a precipitate from the blood exudation and the irritation of constitutional origin*, the disease, if these analyses are confirmed by subsequent experiments, being but another phase of the uric acid or gouty diathesis. These deposits were examined by Professor Ernest Congdon, of the Drexel Institute, whose experimental skill is a sufficient guarantee for the accuracy of the results obtained, and subsequently repeated by Professor A. P. Brubaker with the same results.

